# Activation of NF-*k*B-Mediated TNF-Induced Antimicrobial Immunity Is Required for the Efficient *Brucella abortus* Clearance in RAW 264.7 Cells

**DOI:** 10.3389/fcimb.2017.00437

**Published:** 2017-10-09

**Authors:** Huynh T. Hop, Alisha W. B. Reyes, Tran X. N. Huy, Lauren T. Arayan, WonGi Min, Hu J. Lee, Man H. Rhee, Hong H. Chang, Suk Kim

**Affiliations:** ^1^Institute of Animal Medicine, College of Veterinary Medicine, Gyeongsang National University, Jinju, South Korea; ^2^College of Veterinary Medicine, Kyungpook National University, Daegu, South Korea; ^3^Institute of Agriculture and Life Science, Gyeongsang National University, Jinju, South Korea

**Keywords:** *B. abortus*, TNF, TNFR-1, ROS, NO, NF-*k*B transcriptional factor

## Abstract

In this study, we explore the regulatory roles of pro-inflammatory cytokine tumor necrosis factor alpha (TNF) in the innate immunity of macrophages against *B. abortus* infection. We show that infection of macrophage with *B. abortus* induces marked expression and secretion of TNF which subsequently binds to TNF receptor 1 (TNFR-1) and activates a downstream signaling cascade of the innate immunity. Blocking of TNF signaling resulted in a notable increase of *B. abortus* survival which was associated with an increase of anti-inflammatory cytokine interleukin 10 (IL-10), a beneficial effector of *Brucella* survival, as well as remarkable decrease of reactive oxygen species (ROS) and nitric oxide (NO), antibrucella molecules. However, surprisingly, the interference of TNF did not show any influence on phagolysosome and cell death events. Furthermore, the transcriptional factor NF-*k*B was found to be a main mediator of TNF signaling when blocking of NF-*k*B pathway drastically suppressed the TNF-induced brucellacidal effect. Taken together, these findings clearly indicate that the immune cascade activated by TNF/TNFR-1 is required for the sufficient resistance to *B. abortus* survival in macrophages.

## Introduction

*Brucella* spp. are facultative intracellular Gram-negative bacteria that cause brucellosis in a variety of mammalian hosts including humans with more than 500,000 new cases annually (Hop et al., 2015). In macrophages, *B. abortus* can install phosphatidylcholine to its outer membrane, and can synthesize cyclic β-1,2-glucans that interact with the lipid rafts, leading to preventing phagolysosome fusion event (Reyes et al., 2017). Vaccination seems to be a predominant manner for the control of infectious diseases; however, there is no 100% efficacious vaccine for brucellosis so far (Pasquevich et al., 2009). Thus, identification of host-bacterial interaction could be useful to design rational approaches for brucellosis elimination (Xavier et al., 2013).

Pro-inflammatory cytokine tumor necrosis factor (TNF) is an important regulator of host responses to microbial challenges (Liew et al., 1990; Ziltener et al., 2016). This cytokine amplifies and coordinates pro-inflammatory signals that lead to the expression of effector molecules resulting in the modulation of the diverse aspects of innate immunity against infection. Especially important for the restriction of initial infection, TNF was demonstrated to elicit expression of chemokines and adhesion molecules that are needed for the recruitment of neutrophils to the infection site (Mizgerd et al., 2001). Furthermore, several studies have shown that recombinant TNF is capable of enhancing T cell proliferation in response to a variety of stimuli as well as promoting upregulation of major histocompatibility complex (MHC) molecules, interferon γ (IFN-γ) production and expression of high-affinity interleukin 2 receptor (IL-2R) (Chatzidakis and Mamalaki, 2010).

In macrophages, TNF was reported to activate nuclear factor kappa-light-chain-enhancer of activated B cells (NF-*k*B) through stimulating NF-*k*B inhibitor, IkBα polyubiquitination that is subsequently responsible for controlling inflammation and antimicrobial immune processes (Simon and Samuel, 2007; Gutierrez et al., 2008; Wang and Hardwidge, 2012). In addition, production of reactive oxygen species (ROS) and nitric oxide (NO) was also demonstrated to be controlled by TNF (Liew et al., 1990; Blaser et al., 2016); however, the actual functions of these effectors in *Brucella* clearance are still debatable (Jiang et al., 1993; Gross et al., 1998).

TNF signaling is multi-faceted that includes two different types of TNF: soluble (sTNF) and membrane-bound (mTNF) and two receptors: TNF receptor 1 and 2 (TNFR-1 and TNFR-2). Binding of either form of TNF to its receptors leads to activation of different signaling cascades. TNFR-1 is expressed in majority of cells and can be triggered by either sTNF or mTNF while TNFR-2 is restricted to immune cells and only triggered by mTNF (Blaser et al., 2016). An early study proved that TNF is non-functional in resistance to avirulent *Brucella* strain (S19) (Jiang and Baldwin, 1993). However, soon after, another study has clearly shown that mice lacking the receptor for TNF resulted in the deficient production of NO and interleukin 12 (IL-12) that led to higher susceptibility to virulent *B. abortus* (Zhan and Cheers, 1998). In addition, another report also proved that TNF was remarkably induced by *Brucella* infection in macrophages (Eskra et al., 2003), suggesting an argument in the role of TNF in brucellacidal immunity. Thus, this study reports the immunological roles of TNF and its downstream signaling in response to *B. abortus* infection that is solely associated with anti-inflammatory cytokine IL-10, NF-*k*B, and antimicrobial effectors such as ROS and NO.

## Materials and methods

### Reagents

TNF siRNA, 2-Amino-5,6-dihydro-6-methyl-4*H*-1,3-thiazine hydrochloride (AMTH), NF-*k*B p105/50, p100/52, p65, Lysosomal-associated membrane protein 1 (LAMP-1) and cathepsin H (CTSH) antibodies were obtained from Santa Cruz Biotechnology, TX, USA. TNF recombinant mouse proteins and the other TNF siRNA construct were obtained from Thermo Fisher Scientific, MA, USA while Texas red-goat anti-rat IgG antibody and Lipofectamine RNAiMAX were purchased from Life Technologies, CA, USA. FITC-conjugated goat anti-rabbit IgG antibody, thenoyltrifluoroacetone (TTFA) and diphenylene iodinium chloride (DPI) were obtained from Sigma-Aldrich, MO, USA.

### Bacterial strain and cell culture

A smooth, virulent *B. abortus* 544 biovar 1 strain was kindly provided by Animal and Plant Quarantine Agency in Korea. *B. abortus* was routinely cultured overnight in Brucella broth (BD Biosciences, USA) at 37°C. The murine macrophage RAW 264.7 cells were grown at 37°C in 5% CO_2_ atmosphere in RPMI 1640 containing 10% heat-inactivated fetal bovine serum (FBS) with or without 100 U/ml penicillin and 100 μg/ml streptomycin.

### Bacterial uptake and intracellular replication assay

These assays were performed as previously reported with few modifications (Lee et al., 2012). Briefly, macrophages maintained in RPMI medium supplemented with 10% FBS were replaced by serum-, antibiotic- free RPMI medium and infected with the virulent strain *B. abortus* at a multiplicity of infection (MOI) of 100. At 15, 30, and 45 min post-infection, the infected cells were washed once with phosphate buffer saline (PBS) and then incubated in RPMI/10% FBS and gentamicin (30 μg/ml) for 30 min to kill extracellular bacteria. Finally, the cells were washed with PBS, lysed with distilled water and plated on Brucella agar for bacterial uptake determination. To monitor intracellular growth, after 1 h of incubation at 37°C, 5% CO_2_, the medium of *B. abortus*-infected macrophages were replaced by RPMI/10% FBS and gentamicin (30 μg/ml). At 2, 24, or 48 h post-infection, the cells were then washed with PBS, lysed with distilled water and plated on Brucella agar.

### RNA interference

RAW 264.7 cells were grown to 50% confluence and transfected with either 10 pmol of siRNAs directed against *Tnf* using Lipofectamine RNAiMAX. The cells were incubated for 24 h at 37°C and 5% CO_2_ prior to the performance of intracellular growth assay or other subsequent experiments. The same concentration of negative control siRNA was used throughout as controls. Knockdown efficiency was quantified using qRT-PCR.

### RNA extraction

The total RNA was isolated from RAW 264.7 cells (uninfected or infected with *B. abortus*) at different time points using a Qiagen RNeasy kit (CA, USA). DNA was removed before final elution of the RNA sample using the Qiagen “On-Column DNase Digestion” protocol.

### qRT-PCR

Real-time PCR analysis was performed as previously described (Gutierrez et al., 2008). Briefly, the mixture of SYBR Green PCR master mix (Applied Biosystems, CA, USA) and different pairs of 10 pM primers (Table 1) were denatured at 95°C for 10 min followed by 40 PCR cycles of 95°C for 15 s, 55°C for 30 s, and 60°C for 32 s. The mRNA expression profiles were normalized with respect to β-actin. Fold increase of each gene was calculated using the *2*^−ΔΔCT^ *method*.

**Table 1:** List of primer sequences used for RT-PCR.

**Gene**	**Common name**	**Forward primer**	**Reverse primer**
*b-actin*	β**-**actin	5′-CGCCACCAGTTCGCCATGGA-3′	5′-TACAGCCCGGGGAGCATCGT-3
*Il1b*	Interleukin 1β	5′-CAACCACACAAGTGATATTC-3′	5′-GGATCCACACTCTCCAGCTG-3
*Il6*	Interleukin 6	5′-TCCAGTTGCCTTCTTGGGAC-3′	5′-GTACTCCAGAAGACCAGAG-3′
*Tnf*	Tumor necrosis factor	5′-CACAGAAAGCATGATCCGCG-3′	5′-CGGCAGAGAGGAGGTTGACT-3′
*Il10*	Interleukin 10	5′-TGGCCCAGAAATCAAGGAGC-3′	5′-CAGCAGACTCAATACACACT-3′
*Rab1*	Rab1	5′-CCTTCAATAACGTTAAACAGT-3′	5′-TAGTCTACTACTTTCTTTGTGG-3′
*Rab5a*	Rab5a	5′-GTACTACCGAGGAGCACAAG-3′	5′-AAGCTGTTGTCATCTGCATAG-3′
*Rab5b*	Rab5b	5′-GACTAGCAGAAGTACAGCCAG- 3′	5′-CAATGGTGCTTTCCTGGTATTC-3′
*Rab7*	Rab7	5′-CCTCTAGGAAGAAAGTGTTGC-3′	5′-TTCTTGACCGGCTGTGTCCCA- 3′
*Rab9*	Rab9	5′-GCCCATGCAGATTTGGGACAC-3′	5′-GCCGGCTTGGGCTTCTTCTGTA-3′
*Rab10*	Rab10	5′-GCCGAATGTTACTAGGGAACAAG-3′	5′-GCCGCCTCCTCCACTGCTGATA-3′
*Rab11*	Rab11	5′-GAGCAGTAGG TGCCTTATTGG-3′	5′-GAACTGCCCTGAGATGACGTA-3′
*Rab14*	Rab14	5′-GCCGGAGCTACTATAGAGGAGCT-3′	5′-GCCGTTCTGATAGATTTTCTTGG-3′
*Rab20*	Rab20	5′-CTGCTGCAGCGCTACATGGAGCG- 3′	5′-CTCCGCGGCAGTACAGGGAGC-3′
*Rab22a*	Rab22a	5′-GCCGACAAGAACGATTTCGTGCA-3′	5′-GCCGACTTCTCTGACATCAGTA-3′
*Rab24*	Rab24	5′-GCGCGGGTGAGCACCGCAGGGC-3′	5′-GCCTCAGACCCCAACCCCAAG-3′
*Rab31*	Rab31	5′-GCCCAGAAAACATTGTGATGGCG-3′	5′-GGCATTCTTCGCGCTGGTCTCC-3′
*Rab32*	Rab32	5′-GCCGAGTATACTATAAGGAAGCTC-3′	5′-GCCCTGGGAAGGACTCTGGCTG-3′
*Rab34*	Rab34	5′-GCAAAGTGACCCCGTGTGGCGGG−3′	5′-GGGCGTCCCGAAGACCACTCGG-3′
*Eea1*	Early endosome antigen 1	5′-GCCCAATGAAGAGTCAGCAAGTC-3′	5′-GCCCACCTTGAGATGCTGGCGC-3′
*Rilp*	Rab-interacting lysosomal protein	5′-CAGGAACAGCTACAGCGCCTCCT-3′	5′-CTGAGGTTGCCGCATCAGGTTC-3′
*Sort1*	Sortilin1	5′-GGGGAGCTGCGGACGGCCTTTTG-3′	5′-GGAGGCGCGGGCGGCGGCGGC-3′
*Lamp1*	Lysosomal membrane glycoprotein 1	5′-GGCCGCTGCTCCTGCTGCTGCTG- 3′	5′-ATATCCTCTTCCAAAAGTAATTG- 3′
*Lamp2*	Lysosomal membrane glycoprotein 2	5′-AGGGTACTTGCCTTTATGCAGAAT-3′	5′-GTGTCGCCTTGTCAGGTACTGC−3′
*Stx2*	Syntaxin 2	5′-TGCCGTGGCAGCGCCTGCCCG-3′	5′-GGTCCCGCATCCCCACCGGC-3′
*Stx3*	Syntaxin 3	5′-GATGACACGGACGAGGTTGAGAT-3′	5′-GTTGTGAGCTGTTCAAGGTCATC-3′
*Stx4a*	Syntaxin 4A	5′-CCCACGAGTTGAGGCAGGGGG-3′	5′-GGCGTGGCCAGGATGGTGACC-3′
*Stx5a*	Syntaxin 5A	5′-CGGGATCGGACCCAGGAGTTC-3′	5′- CAAAGAGGGACTTGCGCTTTG-3′
*Stx6*	Syntaxin 6	5′-GTCAACACTGCCCAAGGATTGTTT-3′	5′- GTTTCATCGAGGTCCTCCAGATCC-3′
*Stx7*	Syntaxin 7	5′-GGAAGCCGGCGAGGTCAGGGTGA-3′	5′- CATTGTGTGATCTTTTGGATGTTAG-3′
*Stx8*	Syntaxin 8	5′-GGGCGGAGACTGCACCATGGCCCC-3′	5′- GTCTTCGATCCCCCTCCAGTTGTG-3′
*Stx11*	Syntaxin 11	5′-GCTTCAAGAATTGTCCAGGAGCT- 3′	5′- ATGGACGTGAGGAAGCGGACGTT 3′
*Stx12*	Syntaxin 12	5′-CCGGTCTCTGCTCACTGTCATGTC-3′	5′-GTGGCTTGGCTGATCCGCTGGATG-3′
*Stxbp1*	Syntaxin-binding protein 1	5′-CGGAGCCCGAAGACTCGAAGAACG-3′	5′-CAGCAGGAGGACAGCATCCTCATG-3′
*Stxbp2*	Syntaxin-binding protein 2	5′-CCTCAGGGGAAGATGGCGCCCTTG-3′	5′-CAACAGGATGACAAGATTCGCATG-3′
*Lyz1*	Lysozyme 1	5′-CTCTCCTGACTCTGGGACTCCTCC-3′	5′-CTGAGCTAAACACACCCAGTCAGC-3′
*Lyz2*	Lysozyme 2	5′-GGCCAAGGTCTACAATCGTTGTG−3′	5′-GCAGAGCACTGCAATTGATCCCA−3′
*HexA*	Hexosaminidase A	5′-GCCGGCTGCAGGCTCTGGGTTTC- 3′	5′-GCGCGGCCGAACTGACATGGTAC- 3′
*HexB*	Hexosaminidase B	5′-CCCGGGCTGCTGCTGCTGCAGGC- 3′	5′- GTGGAATTGGGACTGTGGTCGATG- 3′
*Hexdc*	Hexosaminidase D	5′-CCACGCCATTTAAGATGAGATTAG-3′	5′-GGCCCTCAGCAGCCTCAGGTGGCC-3′
*Gla*	Galactosidase, α	5′-GGCCATGAAGCTTTTGAGCAGAG- 3′	5′- AGTCAAGGTTGCACATGAAACGTT- 3′
*Glb1*	Galactosidase, β1	5′-GGAGGTGCAGCGGCTGGCCAGAGC-3′	5′-GGTGACATTATAGATGCCGTGCGC-3′
*Glb1l*	Galactosidase, β1 like	5′-GTGACGGGTGGGAAAGCCCTCACC-3′	5′-CTGTCATGTTCCCGATCCACAACG-3′
*Lpl*	Lipoprotein lipase	5′- CAGACATCGAAAGCAAATTTGCCC-3′	5′- GTCCATCCATGGATCACCACGAAG-3′
*CtsA*	Cathepsin A	5′-GCCCTCCCCGGCCTGGCCAAGCAG-3′	5′-GCCGGCTGGATCAGAAAGGGGCCG-3′
*CtsB*	Cathepsin B	5′-GCCGTGGTGGTCCTTGATCCTTCTT-3′	5′-GCCCCTCACCGAACGCAACCCTTC-3′
*CtsC*	Cathepsin C	5′-GCCGCCACACAGCTATCAGTTACTG-3′	5′-GCCCCTGGAGACCTCCAAGATGTGC-3′
*CtsD*	Cathepsin D	5′-CGTCTTGCTGCTCATTCTCGGCCTC- 3′	5′-CACTGGCTCCGTGGTCTTAGGCGAT- 3′
*CtsE*	Cathepsin E	5′-GGAGCAGAGTGAGAGAGAAGCTAC-3′	5′- GGGCCCGTAGTTTCTTCCGAAGGG-3′
*CtsF*	Cathepsin F	5′-GCC GCA GGC TCC GCC TCG-3′	5′-GCC GCT CCT AGC ACG GCC-3′
*CtsG*	Cathepsin G	5′- CCTGTGCACACCTGTATCTACATAA-3	5′- CTGTGTACCGAGTCACCGTACACGC-3′
*CtsH*	Cathepsin H	5′- CTGAGAACCCTTCTTCCCAAGAGC−3′	5′- AGCAGCCAGGCCCCAGCGCACAGC−3′
*CtsK*	Cathepsin K	5′- GGATGAAATCTCTCGGCGTTTAAT-3′	5′- GTCTCCCAAGTGGTTCATGGCCAG-3′
*CtsL*	Cathepsin L	5′-GCCCCTTTTGGCTGTCCTCTGCTT-3′	5′-GCCCTCCATGGAAAAGCCGTGC-3′
*CtsO*	Cathepsin O	5′-GCCCGCAGTTGGTGAACCTCTTGCT-3′	5′-GCCGTCCTTCTGCTGGGTATCTGGG-3′
*CtsS*	Cathepsin S	5′-GCCGACTACCATTGGGATCTCTGGA-3′	5′-GCCGTCTCCCATATCGTTCATGCCC-3′
*CtsZ*	Cathepsin Z	5′- GGCGTCGTCGGGGTCGGTGCAGCA- 3′	5′- CTGCGCCCCAGCAGAGCCAGCTG- 3′
*Man1a*	Mannosidase 1, α	5′- CAAGCTGCTCAGCGGGGTCCTGTT- 3′	5′-GCGGATCCTGGCTAAGTTGTCTTC- 3′
*Man1a2*	Mannosidase 1, α2	5′- GAAACTAGGTCCGGAGTCATTCAAG-3′	5′-CTTCCCAGCCCCACTGCCTGTATC-3′
*Man2a1*	Mannosidase 2, α1	5′- GCTACA GACATTTTGT GCCATATG-3′	5′- CTGGGGGAACTCCCCAGGGACAAC-3′
*Man2a2*	Mannosidase 2, α2	5′-GGATAGAACAGCTGGAACAA CTGC-3'	5′- CCCCGTCCCCCCAAAGCAAACTGG-3′
*Man2b1*	Mannosidase 2, α B1	5′- G TGATGTTCAG CACGCATCTG TTC-3′	5′- CGTACAGCGTCCTGGGTTGCACTG-3′
*Man2b2*	Mannosidase 2, α B2	5′- CCGTCTTCCC AGAGCCACCC CCAG-3'	5′- CAGAGGACGTGGGGCGTCCGGAAC-3'
*Man1c1*	Mannosidase α Class 1C Member1	5'- GAGGCCATAG AGACCTATCT CGTG-3′	5′- CATGGCACGTCCTGGTGATCTGGG-3′
*Man2c1*	Mannosidase α Class 1C Member1	5′- GTAGCCTGCA ATGGGCTTCT GGGG-3′	5′- CAACAGCTCCAGGTCCACCAGGAG-3′

### Collection of cytoplasmic and nuclear protein extracts from macrophages

The cytoplasmic and nuclear protein extracts were obtained as previously described (Maribel et al., 2013). Briefly, the uninfected or infected macrophages were collected, suspended in buffer A (10 mM HEPES, pH 7.9, 10 mM KCl, 1.5 mM MgCl_2_, 1 mM DTT) and then frozen in a dry ice-acetone bath. Afterwards, they were thawed in ice bath and the cytoplasmic extracts were collected as supernatants after centrifugation at 1200 × g at 4°C for 10 min. The pellet containing the nuclei was resuspended in buffer C (20 mM HEPES, pH 7.9, 0.4 M NaCl, 1.5 mM MgCl_2_, 25% glycerol, 0.2 mM EDTA, 1 mM DTT), supplemented with 0.5 mM phenylmethylsulfonyl fluoride (PMSF) proteases inhibitor (Sigma-Aldrich, CA, USA). This mixture was incubated under gentle stirring and then centrifuged at 20,000 × g at 4°C for 20 min. The supernatant was collected as the nuclear extract. Buffer D (20 mM HEPES, pH 7.9, 50 mM KCl, 25% glycerol, 0.2 mM EDTA, 1 mM DTT), supplemented with 0.5 mM PMSF proteases inhibitor was added to the cytoplasmic and nuclear extracts which were stored and maintained at −70°C until needed.

### Western blot assays

The lysates of cells were identified by Western blot assay as previously described (Hop et al., 2015). Briefly, the proteins were boiled for 5 min at 100°C in 2x SDS buffer. After electrophoresis, separated proteins were transferred onto Immobilon-P membranes (Milipore, MA, USA) for 60 min using a semi-dry electroblot assembly (Bio-Rad, CA, USA). The membranes were blocked with 5% skim milk (Thermo Scientific, MA, USA) and incubated with primary antibodies (1:1000 dilution) in blocking buffer. The membranes were then incubated with horseradish peroxidase (HRP)-conjugated goat anti-mouse IgG antibody (1:1000 dilution) in blocking buffer. The proteins were detected with enhanced chemiluminescence (ECL) solution (Thermo Scientific, MA, USA).

### LAMP-1 and CTSH staining

The colocalization of *Brucella*-containing phagosomes (BCPs) with these markers was performed as previously reported (Kim et al., 2004; Lee et al., 2013). Briefly, after 1 h of incubation in RPMI/10% FBS containing gentamicin (30 μg/ml), RAW 264.7 cells were fixed with 4% paraformaldehyde, permeabilized with 0.1% Triton X-100 and blocked with blocking buffer (2% goat serum in PBS). The samples were stained with anti-LAMP-1 or anti-CTSH antibodies at dilution of 1:100 in blocking buffer. The samples were subsequently stained with Texas red-goat anti-rat IgG (1:100) in blocking buffer followed by staining with anti-*B. abortus* rabbit serum and FITC-conjugated anti-rabbit IgG to identify the bacteria and placed in mounting media. Fluorescence images were captured using a laser scanning confocal microscope (Olympus FV1000, Japan) and processed using FV10-ASW Viewer 3.1 software. The percentage of these proteins colocalization was determined from 100 random cells.

### Indirect immunofluorescence

This assay was performed as previously described (Gutierrez et al., 2008). Briefly, the cells were fixed with 4% paraformaldehyde, permeabilized with 0.1% Triton X-100 and incubated with blocking buffer (2% goat serum in PBS). Then, rabbit anti-p52 and p65 antibodies (1:100 dilution) were added and followed by incubation with Texas red or FITC-conjugated goat anti-rabbit IgG antibody (1:100 dilution). The nucleus was stained and cells mounted with Prolong Gold Antifade Reagent with DAPI (Thermo Fisher, MA, USA) and incubated at room temperature for 30 min. The cells were then analyzed by laser scanning confocal microscope (Olympus FV1000, Japan) and processed using the FV10-ASW Viewer 3.1 software.

### ELISA

TNF, IL-6, IL-10, and IL-1β levels in the culture supernatants were determined by ELISA in accordance with the manufacturer's instructions (ThermoFisher Scientific, MA, USA).

### ROS detection

*B. abortus*-infected RAW 264.7 cells were treated with either TNF siRNAs, rTNF, TTFA, or DI. At 24 h post-infection (pi), ROS content was evaluated by fluorescence microscopy (Enzo Life Sciences, NY, USA) and spectrometry (Abcam, Cambridge, USA) according to the manufacturer's instructions.

### NO and nitrite detection

RAW 264.7 cells were treated with either TNF siRNA or rTNF and then infected with *B. abortus* for 24 h. Cellular NO content was evaluated by fluorescence microscopy (Enzo Life Sciences, NY, USA). The nitrite concentration from culture supernatant was also assessed by Griess reaction (Sigma-Aldrich Corp, Missouri, USA).

### Flow cytometry for apoptosis and necrosis

The apoptosis and necrosis were evaluated by flow cytometry using apoptosis and necrosis detection kit (Abcam, Cambridge, USA) at 48 h post-infection (pi) in accordance with the manufacturer's instructions. Briefly, after 2 days incubation at 37°C, infected macrophages were stained with apoptosis indicator (apopxin green) and necrosis indicator (7-AAD). The apoptosis and necrosis percentages were determined from 10,000 random cells.

### Statistical analysis

The data are expressed as the mean ± standard deviation (*SD*). ANOVA with Tukey's HSD exact test was used to statistically compare the groups. Results with *P* < 0.05 were considered significantly different.

## Results

### TNF/TNFR1 signaling contributes to *B. abortus* clearance in raw 264.7 cells

**Figure 1:**
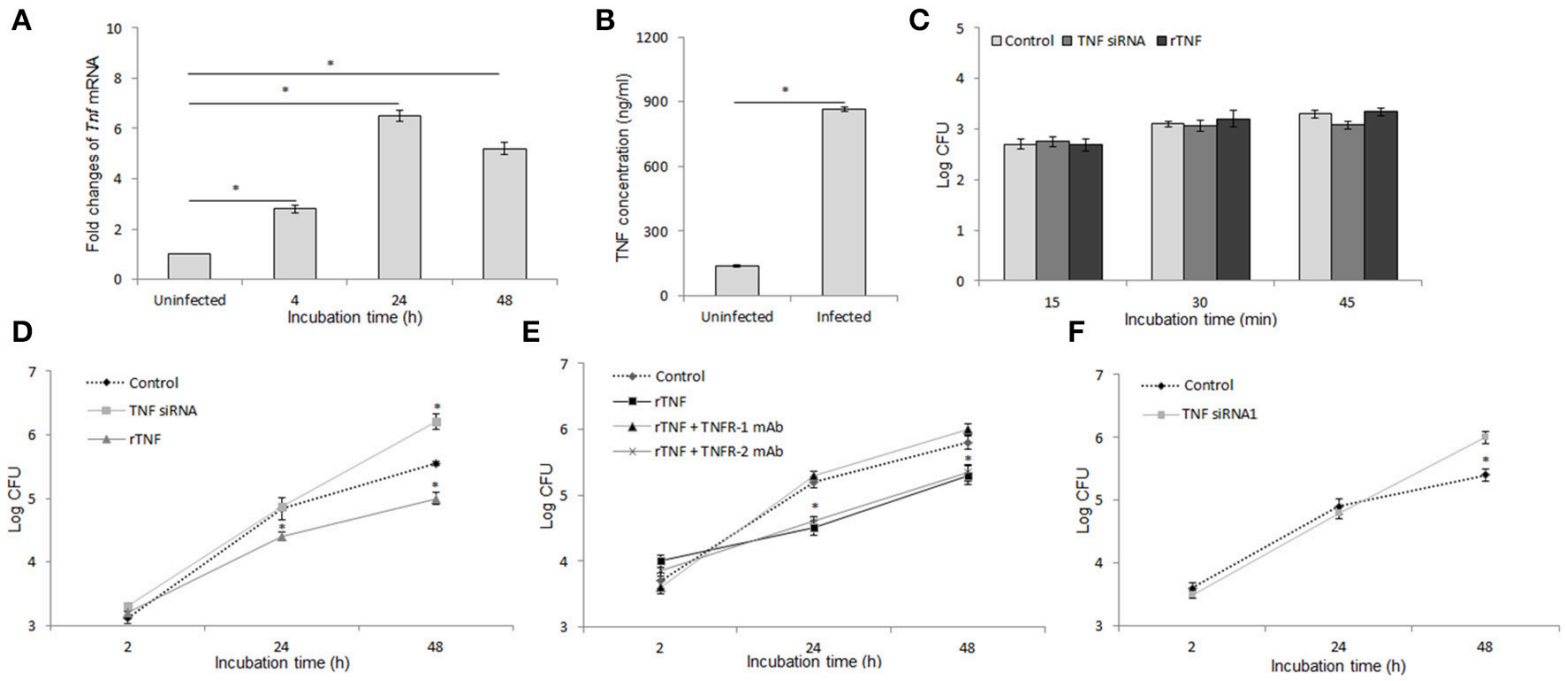
TNF activates antibrucella immunity through binding with TNFR-1. Macrophages infected with *B. abortus* and the transcriptional and translational profiling of *Tnf* were examined by qRT-PCR **(A)** and sandwich ELISA **(B)**. Bacterial uptake **(C)** and intracellular growth **(D)** were evaluated in cells with or without treatment of either TNF siRNA or rTNF. The cells were concomitantly treated with rTNF and either anti-TNFR-1 or -TNFR-2 antibodies, and the intracellular growth of *B. abortus* was assessed **(E)**. The validation of TNF suppression was performed by cells treated with TNF siRNA1 and survival rate of bacteria were evaluated **(F)**. Data represent the mean ± *SD* of triplicate experiments. Asterisk indicated the significant difference (*P* < 0.05).

Previous study has shown that TNF is induced by *Brucella* infection in macrophages and plays a critical role in resistance to *Brucella* infection *in-vivo* (Zhan and Cheers, 1998; Eskra et al., 2003), suggesting that TNF is also a key effector in innate immunity against *Brucella* infection in macrophages. To address this hypothesis, we first evaluate the impact of *B. abortus* infection in the production of TNF in RAW 264.7 cells. Interestingly, by using qRT-PCR to analyze the transcriptional profile of this cytokine at different time points, we found that *B. abortus* markedly induces *Tnf* mRNA as early as 4 h pi and peaked with fold increase of ~6.5 times at 24 h pi compared to control (Figure 1A). Additionally, the quantification from culture supernatant by ELISA at 48 h pi was also shown to be consistent with the high induction of secreted TNF by *B. abortus* infection (Figure 1B).

To examine the role of TNF in the anti-microbial immunity, macrophages were treated with either specific TNF siRNA or rTNF and infected with *B. abortus*. Bacterial CFU was determined at indicated times. As shown in Figure 1C, interference of TNF signaling did not influence bacterial internalization; however, the inhibition of TNF pathway caused a significantly increased survival of intracellular *Brucella* while the reverse result was obtained when rTNF was supplied (Figure 1D). The TNF signaling is known to be mediated by TNFR-1 and TNFR-2 which subsequently activate different signaling cascades (Blaser et al., 2016). Thus, to clarify which receptor is the mediator in this context, we concomitantly treated *B. abortus*-infected macrophages with rTNF and either anti-TNFR-1 or -TNFR-2 antibodies. Significant suppression of rTNF-induced *Brucella* clearance was observed in TNFR-1 blocked cells whereas no difference was found in TNFR-2 antibody treatment (Figure 1E), indicating that the bactericidal effect of TNF is solely mediated by TNFR-1.

To avoid the off-target effect of siRNA treatment, we further validated the above findings by using the other TNF siRNA (TNF siRNA1) that target differently on Tnf mRNA sequence. As a prevailing logic, the results were also shown to be of the same trend with the other one (Figure 1F), indicating that our findings were the real effect of TNF knock-down. Taken together, these data clearly indicated the activation of *B. abortus*-induced TNF/TNFR-1 which is required for an efficient killing of *B. abortus* by macrophages.

### Targeting IL-10 restores the killing capacity of TNF blocking cells

**Figure 2:**
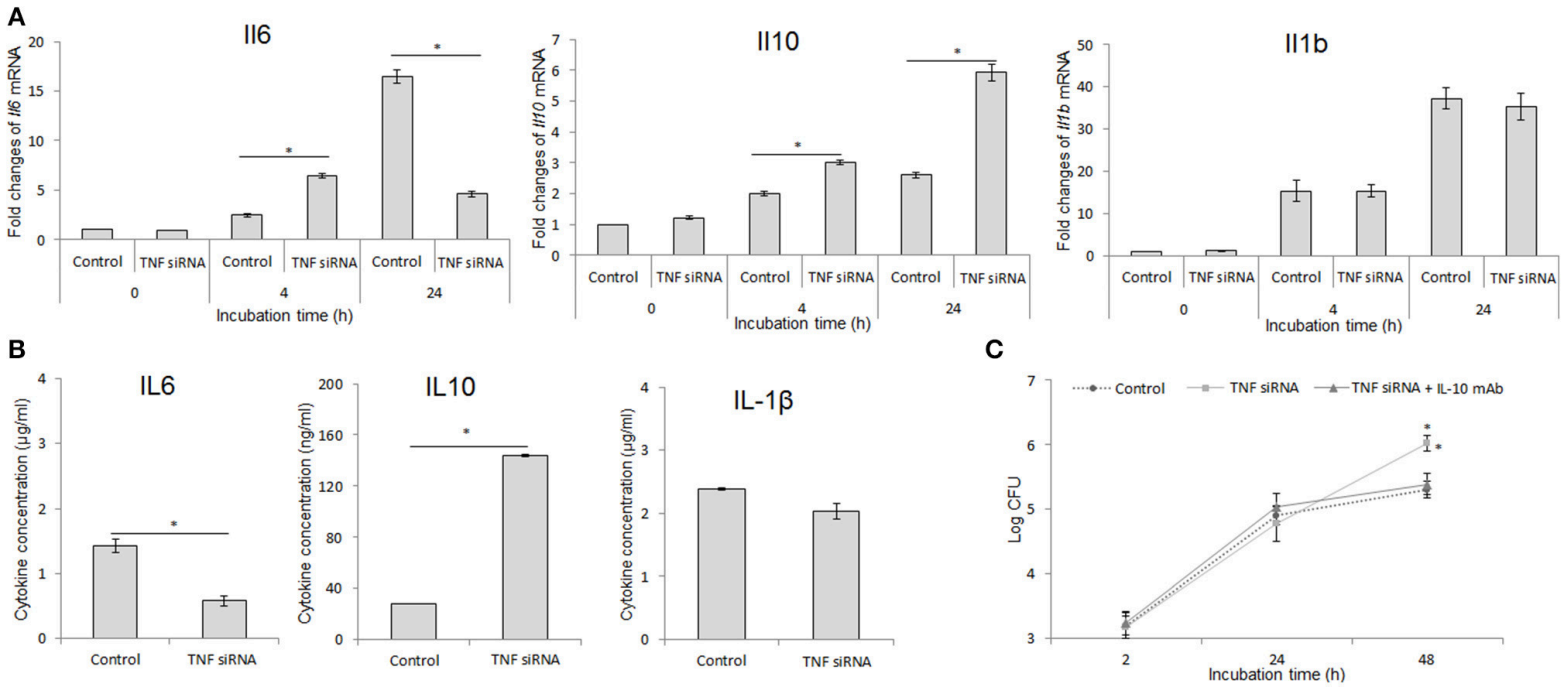
Inhibition of IL-10 signaling significantly restored the antimicrobial effect in TNF-lacking cells. Macrophages were treated with TNF siRNA prior to *B. abortus* infection. Total RNA content was isolated and the expressions of inflammatory cytokines including *Il6, Il10*, and *Il1b* were evaluated by RT-PCR at different time points **(A)**. The presence of secreted cytokines was also checked by Western blot at 48 h pi **(B)**. The TNF-blocking cells were infected with *B. abortus* and treated with anti-IL-10 antibody. The intracellular growth of *B. abortus* within macrophages was then evaluated **(C)**. Data represent the mean ± *SD* of triplicate experiments. Asterisk indicated the significant difference (*P* < 0.05).

Given higher degree of *B. abortus* replication in TNF-suppressive macrophages and the known influence of inflammatory cytokines on macrophage function (Fernandes and Baldwin, 1995; Arango Duque and Descoteaux, 2014), we investigated the regulation of TNF on the inflammatory cytokine expression. For this, the cells were treated with either TNF siRNA or rTNF and the transcripts including *Il6, Il10*, and *Il1b* were assessed by qRT-PCR at 24 h pi. Intriguingly, the level of *Il6* was highly suppressed while *Il10* was remarkably increased in *B. abortus*-infected macrophages lacking TNF, and the reverse results were obtained with cells supplemented with rTNF. However, the expression of *Il1b* was shown to be independent with TNF signaling (Figure 2A). To complement these data, we further quantified the presence of these cytokines in culture supernatant at 48 h pi by ELISA. Consistent with qRT-PCR result, the presence of IL-6, IL-10, and IL-1β was shown to be decreased, increased and unchanged, respectively, in TNF-suppressive cells compared to control (Figure 2B). These data argue that the higher survival of intracellular *B. abortus* in TNF blocking macrophages may be due to the shift of the immune response toward the anti-inflammatory phenotype characterized by increased IL-10 level. To figure out this assumption, we concomitantly treated cells with TNF siRNA and anti-IL-10 antibody. As predicted, we found that high production of IL-10 mainly contributes to poor control of *B. abortus* in TNF blocking cells since neutralizing IL-10 by specific antibody substantially restored the killing capacity of *B. abortus* in TNF-blocking macrophages (Figure 2C). Altogether, our data suggest that the pro-inflammatory antibrucella effect of TNF is through suppression of IL-10 signaling which is beneficial to *Brucella* persistence in macrophages.

### TNF stimulates mitochondrial ROS and NO production as crucial effectors of *B. abortus* clearance in macrophages

**Figure 3:**
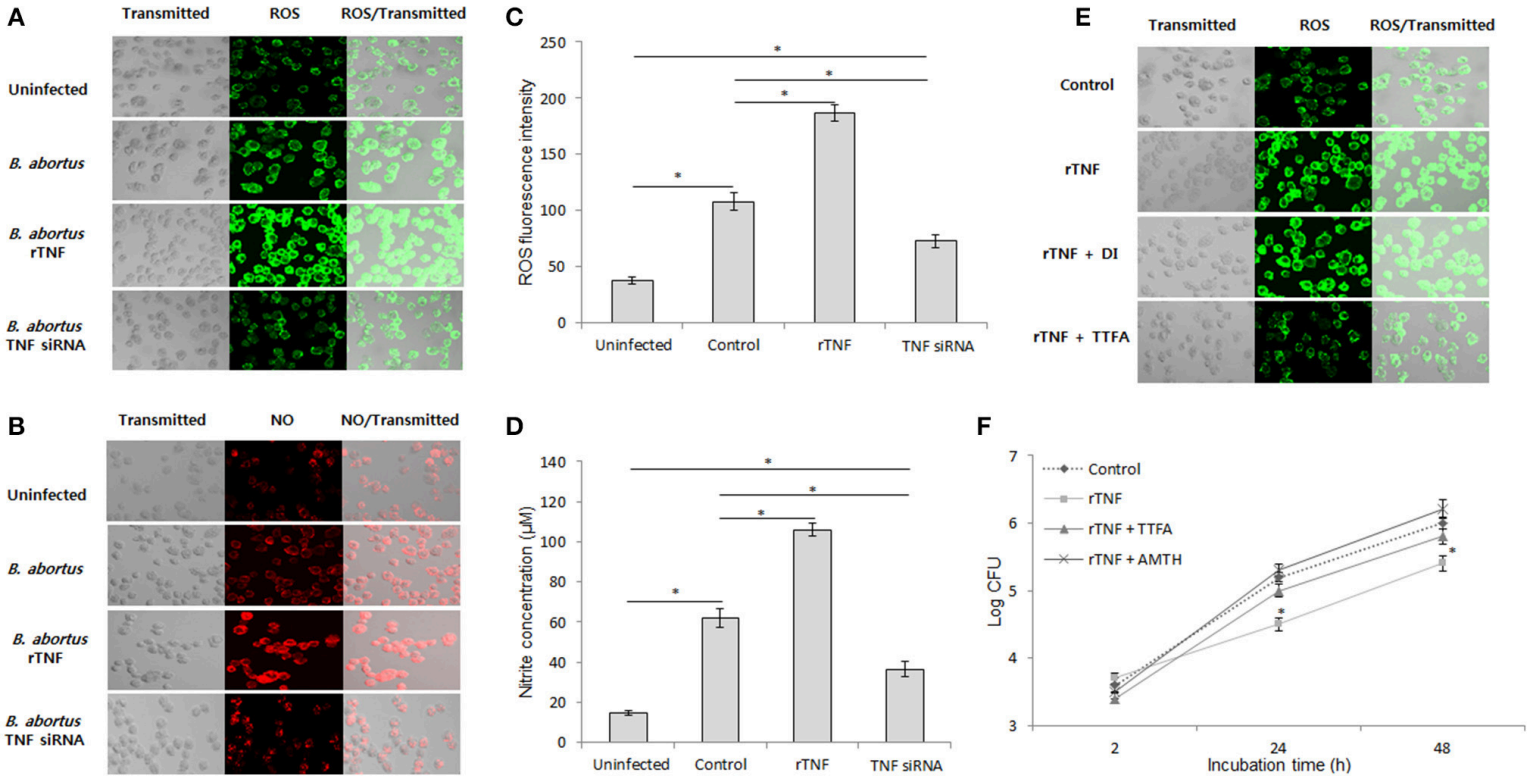
TNF mediates mitochondrial ROS and NO to control intracellular *B. abortus*. Macrophages were treated with TNF siRNA prior to *B. abortus* infection. The accumulation of ROS **(A)** and NO **(B)** was evaluated by fluorescence microscopy. The cellular ROS **(C)** and the nitrite concentration in culture supernatant **(D)** were further confirmed at 24 h post-infection by spectrometry assay and Griess reaction, respectively. To determine the source of ROS, the *Brucella*-infected cells were treated with either mitochondrial inhibitor (TTFA) or NADPH inhibitor (DPI), and the ROS production was checked by fluorescence microscopy at 24 h pi **(E)**. The role of ROS and NO in TNF-induced protective immunity were examined by treatment of *Brucella*-infected cells with either TTFA or NOS2 inhibitor (AMTH). The intracellular growth of *B. abortus* was then evaluated **(F)**. Data represent the mean ± *SD* of triplicate experiments. Asterisk indicated the significant difference (*P* < 0.05).

ROS and NO are important effectors that serve for host defense against infectious agents by direct and indirect activities (Liew et al., 1990; Gross et al., 1998; Deffert et al., 2014). In the mouse model, knockout of either cytochrome B-245 beta chain (*gp91*^*phox*^) or inducible NO synthase (*Nos2*) were shown to markedly enhance the persistence of *Brucella* (Ko et al., 2002), suggesting their essential roles in brucellacidal activity. Thus, we examined the relation of TNF signaling and intracellular ROS and NO production in responses to *B. abortus* infection in macrophages by fluorescence microscopy. We found that the intracellular levels of both ROS and NO were significantly decreased when TNF signaling was inhibited and enhanced when rTNF was supplied (Figures 3A,B). Further confirmation with spectrometry (ROS) and Griess reaction (nitrite) also showed reduction of these chemically-reactive molecules by TNF siRNA treatment (Figures 3C,D), suggesting the potential involvement of ROS and NO in TNF-mediated signal transduction in *Brucella*-infected macrophages. To further identify the source of TNF-induced intracellular ROS accumulation in infected RAW 264.7 cells, we concomitantly treated rTNF with either TTFA or DPI which are inhibitors of mitochondrial respiratory chain and NADPH oxidase (NOX), respectively. As shown in Figure 3E, only treatment with TTFA resulted in a remarkable decrease of rTNF-induced ROS accumulation, indicating that TNF stimulates ROS production through controlling mitochondrial respiratory chain function. In parallel, the treatment of either TTFA or AMT hydrochloride (AMTH), a selective inhibitor of NOS2, also repressed the rTNF-induced *Brucella* clearance (Figure 3F), suggesting that mitochondrial ROS and NO are key effectors of TNF-mediated killing of *Brucella* in murine macrophages.

### Interference of TNF signaling did not influence the phagolysosome fusion and cell death processes during *B. abortus* infection

**Figure 4:**
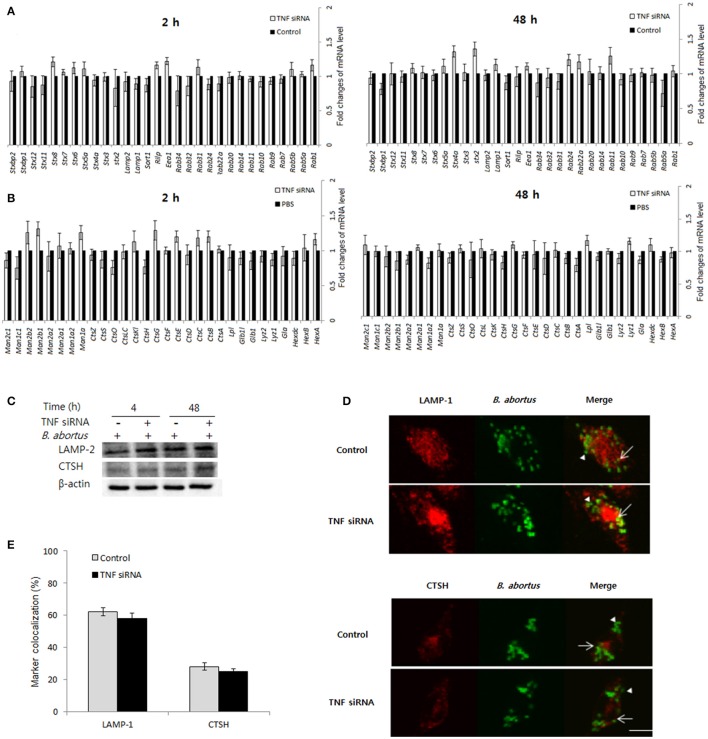
The protective immune responses activated by TNF/TNFR-1 are independent with phagolysosome fusion. Macrophages were treated with TNF siRNA prior to *B. abortus* infection. The total RNA content was isolated, and the expression levels of representative trafficking regulators **(A)** and lysosomal enzymes **(B)** were evaluated by qRT-PCR at 2 and 48 h pi. The total protein extracts were also collected and the expression of representatives was examined by Western blot at 2 and 48 h pi **(C)**. The colocalization of BCPs with LAMP-1 and CTSH was analyzed at 2 h pi **(D)**. Marker positive (arrows) or negative bacteria (arrow heads) were visualized by fluorescence microscopy. The percentage of marker colocalized with BCPs in 100 cells was determined **(E)**. Data represent the mean ± *SD* of triplicate experiments. Scale bars = 5 μm.

Phagolysosome fusion occur as the most important event in the killing of intracellular pathogens (Kim et al., 2004; Gutierrez et al., 2008), thus we hypothesized whether interference of TNF signaling could also alter this process. In an attempt to figure out this functional relationship, the different transcripts including trafficking regulators and hydrolytic enzymes were assessed by qRT-PCR when TNF signaling was blocked. Interestingly, the expression of 60 representatives was shown to be independent with TNF signaling (Figures 4A,B). Further checking by Western blot (Figure 4C) and confirmation of the colocalization by fluorescence microscopy (Figures 4D,E) was also shown to have no difference between control and TNF-lacking cells.

**Figure 5:**
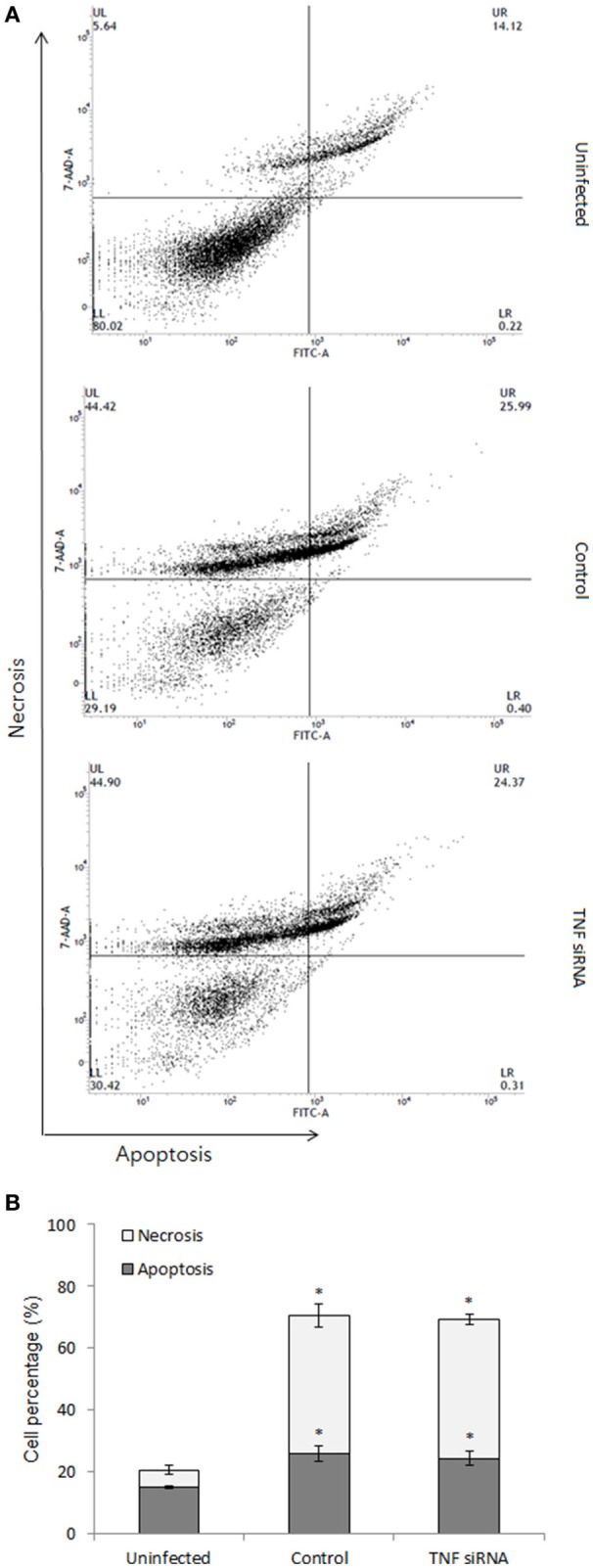
Cell death assay was carried out at 48 h pi. The infected cells were stained with apoptosis indicator (apopxin green) and necrosis indicator (7-AAD) and subjected to analysis by flow cytometry **(A)**. The percentage of apoptotic and necrotic cells were presented **(B)**. Data represent the mean ± *SD* of triplicate experiments. An asterisk indicates a significant difference (*P* < 0.05).

On the other hand, the death of infected cells was demonstrated to play importantly in the outcome of bacterial infection (Jorgensen et al., 2017) and TNF is known as a potent stimulator of cell death such as apoptosis and necrosis. Thus, we tested the effect of TNF on the cell death process in *B. abortus*-infected macrophages. For this, the cells were treated with TNF siRNA prior to infection with *B. abortus* (MOI: 100) which was followed by staining with Apopxin and 7-ADD indicators and finally evaluated at 48 h pi by flow cytometry. Infection with *B. abortus* induced ~10 and 30% of apoptosis and necrosis, respectively; however TNF signaling did not contribute to these processes when no difference on cell death rate was obtained between TNF-suppressive and normal cells (Figures 5A,B).

For further validation of these data, we treated *B. abortus*-infected macrophages with recombinant TNF (rTNF). We next evaluated the expression of 20 representatives by qRT-PCR at 2 h post-infection and cell death by flow cytometry at 48 h post-infection. As predicted, the expression of representatives and the cell death were not influenced by the presence of additional TNF (data not shown).

Altogether, our results suggest that phagolysosome fusion event and cell death processes are not downstream effectors of TNF-induced brucellacidal effect in macrophages.

### TNF induces brucellacidal immunity through activating NF-*k*B pathway

**Figure 6:**
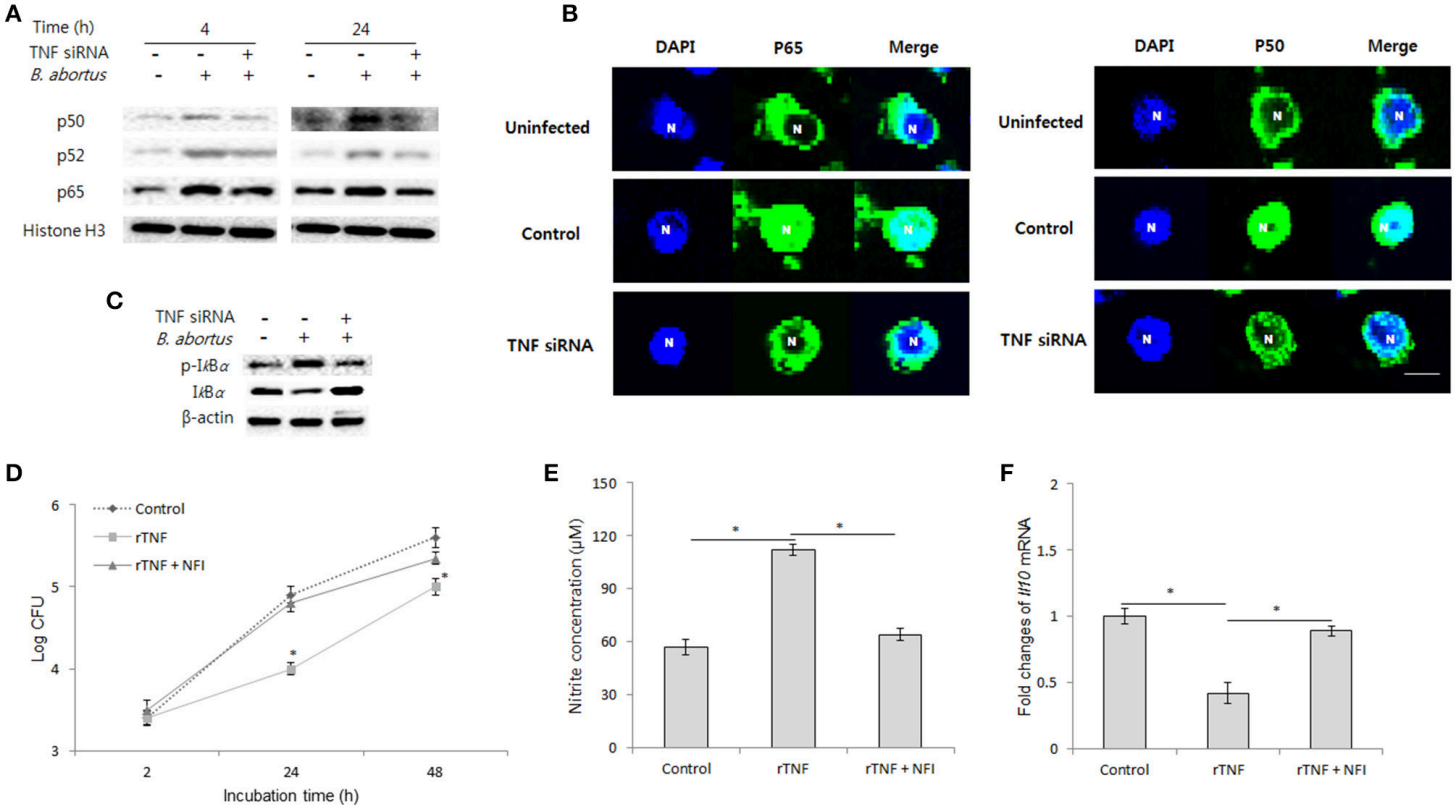
NF-*k*B plays a major role in TNF/TNFR-1 signaling during *B. abortus* infection. Infected RAW 264.7 cells were treated with either recombinant TNF (rTNF) or TNF siRNA and subjected to isolation of nuclear protein fractions. The activation of NF-*k*B proteins was determined by Western blot assay **(A)**. Further observation of NF-*k*B translocation was performed by fluorescence microscopy **(B)**. The phosphorylation level of I*k*Bα was checked by Western blot from total cellular protein extraction **(C)**. To examine the actual role of transcriptional factor NF-*k*B in TNF signaling, the cells were concomitantly treated with rTNF and NF-*k*B translocation inhibitor (NFI), and the intracellular growth of *Brucella* was evaluated **(D)**. The concentration of nitrite **(E)** in culture supernatant and transcriptional level of *Il10* **(F)** was checked by Griess reaction and qRT-PCR at 24 h pi, respectively. Data represent the mean ± *SD* of triplicate experiments. An asterisk indicates a significant difference (*P* < 0.05).

The binding of TNF to TNFR-1 can modulate NF-*k*B activation, a key transcriptional factor that drives cells to different subsequent signaling (Blaser et al., 2016). Thus, the question as to whether TNF mediates NF-*k*B pathway for controlling intracellular *B. abortus* was raised. For this, we blocked TNF signaling by siRNA and conducted activation analysis of NF-*k*B using immunoblotting for nuclear protein extracts at 4 and 24 h pi. Interestingly, inhibition of TNF signaling drastically reduced the translocation of p65 and p50 but not p52 as early as 4 h until 24 h pi (Figure 6A). Moreover, similar data were also observed microscopically (Figure 6B); suggesting that TNF/TNFR-1 signaling controls NF-*k*B activation during *B. abortus* infection. In addition, NF-*k*B activation are mainly controlled by I*k*B proteins, thus as a prevailing logic, we next focused on the analysis of phosphorylation of I*k*Bα, the most important known regulator of NF-*k*B pathway (Wang and Hardwidge, 2012). As predicted, we found that inhibition of TNF signaling notably decreased the level of phosphorylated I*k*Bα compared to the control (Figure 6C), indicating TNF mediates I*k*Bα to control NF-kB pathway.

To validate the actual role of NF-*k*B in the antibrucella effect of TNF, we concomitantly treated infected cells with rTNF and NF-*k*B inhibitor (NFI). As expected, the inhibition of NF-*k*B markedly repressed the induction of *Brucella* clearance by rTNF (Figure 6D). Furthermore, in parallel, treatment of NFI also resulted in a marked decrease of NO production and increase of IL-10 expression as compared to rTNF-treated cells (Figures 6E,F). Taken together, our findings indicated that TNF induces antibrucella immunity through activation of the key transcriptional factor, NF-*k*B.

## Discussion

*B. abortus*, a causative agent of brucellosis, is one of the pathogens which have acquired the ability to survive and replicate within host cells by mechanisms that are still yet to be elucidated. Thus, this study is aimed to provide new evidences in the host-*Brucella* interaction by investigating the immune signaling pathways that are activated by pro-inflammatory cytokine TNF during *B. abortus* infection in macrophages.

In agreement with previous observations (Zhan and Cheers, 1998; Eskra et al., 2003), we showed here that *B. abortus* infection markedly induces expression and secretion of TNF that, in turn, binds to TNFR-1 and activates one of the most important mechanisms to resist intracellular *Brucella* survival. This immune activation was demonstrated to be directly involved in the inflammation activity since inhibition of TNF signaling led to a remarkable increase of IL-10 and a drastic decrease of IL-6 as early as 4 h and continuously until 24 h pi. The anti-inflammatory cytokine, IL-10 was most known to support the persistence of *Brucella* within phagocytes (Fernandes and Baldwin, 1995; Xavier et al., 2013); thus, the increase of IL-10 in this context is suspected to be responsible for the enhanced number of bacteria. On the other hand, lysosome-mediated killing is one of most important defense mechanism against microbial infection. Previous works have shown that IL-10 controls this process through functioning on a variety of trafficking regulators and hydrolytic enzymes (Xavier et al., 2013; our unpublished data). In this report, we showed that TNF signaling is dependent, at least in part, in IL-10 function since neutralizing IL-10 restored *Brucella* killing in TNF-lacking cells. However, we also surprisingly found that TNF signaling is not associated with the phagolysosome fusion event when interference in TNF signaling did not change the recruitment of regulators and lysosomes to BCPs. These data suggest that TNF may have a unique role in regulating the phagolysosome fusion-independent pathway of IL-10 during *B. abortus* infection.

The respective contribution of NO to antibrucella immunity is controversial. iNOS^−/−^ mice were reported to be highly susceptible to *Brucella* and NO was also identified as an important molecule in killing of *Brucella* (Gross et al., 1998; Grillo et al., 2012); however, another report has shown that NO is not sensitive to *Brucella*, because *Brucella* has mechanisms to prevent NO from entering bacterial cell and detoxifying NO (Jiang et al., 1993). We herein report that inhibition of TNF reduced NO production that paralleled with the decreased survival of *Brucella*. In addition, blocking of NO also inhibited the protective immunity against *Brucella* by rTNF. These data clearly indicated the essential role of NO in the innate immune system against *Brucella* infection, as well as in defense mechanism activated by TNF in macrophages.

To date, there are pieces of evidence that ROS, a byproduct of oxygen metabolism, enables to inhibit the propagation of a variety of intracellular bacteria through incompletely understood mechanisms (Paiva and Bozza, 2014). ROS is thought to play its roles through two distinct pathways: causing oxidative damage on bacterial biocompounds and affecting the signaling control of cytokine, autophagy and apoptosis (Jiang et al., 1993; Simon et al., 2000). In this study, we found that binding of TNF to its receptor, TNFR-1 induces a remarkable ROS production in *Brucella*-infected macrophages at late infection through upregulating the function of mitochondrial chain. The treatment of mitochondrial chain inhibitor (TTFA) did not only suppress the production of ROS, but also led to increase intracellular *Brucella*, suggesting that this molecule could be an essential effector of host immunity against *B. abortus* infection. On the other hand, a number of previous works (Simon et al., 2000; Blaser et al., 2016; Jorgensen et al., 2017) reported the induction of cell death through mitochondrial ROS increase; however, our data clearly indicated the independent pathways of TNF-induced mitochondrial ROS and programmed cell death (apoptosis and necrosis) in *B. abortus*-infected macrophages, leading to the argument of other unknown mechanisms activated by *B. abortus* to inhibit cell death inductive function of TNF.

A key question in this study is how TNF suppresses anti-inflammation and induces production of ROS and NO. In this report, we have demonstrated the direct link between TNF and NF-*k*B activation in controlling antibrucella immunity. The transcriptional activity of NF-*k*B is tightly regulated at multiple steps in immune signaling pathways in which one mechanism to prevent NF-*k*B activation is mediated by I*k*B family proteins that traps NF-*k*B in the cytoplasm (Yamamoto and Takeda, 2008). Among I*k*B family proteins, I*k*Bα is known as the most powerful regulator for cRel-p50, p65-p50 and p65-p52 heterodimers (Nishikori, 2005). Upon microbial infection or stimulation by cytokines such as TNF, IkBα is mainly phosphorylated by IKKα and IKKβ, leading to ubiquitination and degradation, and results in the activation and transcriptional activity of NF-kB (Lavon et al., 2000; Adli et al., 2010). We here showed that in the context of *B. abortus* infection, binding of TNF to TNFR-1 stimulated IkBα phosphorylation that led to the activation of classical NF-*k*B pathway. In addition, the inhibition of NF-*k*B significantly decrease the induction of brucellacidal effect by rTNF which paralleled with the decrease of NO production and pro-inflammatory cytokine IL-6 whereas increase of IL-10 production, suggesting an undescribed role of NF-*k*B in controlling antimicrobial effectors induced by TNF signaling. Although we have not explored the interaction between TNF/TNFR-1-activated NF-*k*B and ROS production, it is possible that NF-*k*B could also cooperate with ROS accumulation during *B. abortus* infection.

In summary, our study uncovers the predominant role of TNF in the development of protective immunity against *B. abortus* infection by macrophages. We proved that shifting cells to inflammation and enhancing chemically-radical effectors are two main manners of TNF/TNFR-1 signaling. We also demonstrated the essential roles of NF-*k*B in antibrucella effect of TNF. Therefore, taken together, our results provide new insights into how a proinflammatory cytokine develops protective immunity against *Brucella* infection in macrophages.

## Author contributions

HH, LA, AR and TH carried out all experiments, contributed to data collection and analysis, and participated in drafting the manuscript. WM, HL, MR and HC participated in the design of the study and contributed to revise the manuscript. SK participated in the design of the study, carried out the data analysis, conceived the experiment and prepared the manuscript. All authors read and approved the final manuscript.

### Conflict of interest statement

The authors declare that the research was conducted in the absence of any commercial or financial relationships that could be construed as a potential conflict of interest.
